# Frequently Underappreciated Considerations in Anti-arrhythmic Drug Therapy

**DOI:** 10.19102/icrm.2026.17026

**Published:** 2026-02-15

**Authors:** James A. Reiffel

**Affiliations:** 1 Department of Medicine, Division of Cardiology, Columbia University, New York, NY, USA

**Keywords:** Anti-arrhythmic combinations, anti-arrhythmic drugs, disopyramide, ranolazine, sotalol

## Abstract

Anti-arrhythmic drugs (AADs) have been a mainstay of dysrhythmia control for over a century. Even in the current era of evolving ablation use and technology, AADs remain therapeutically important. Nonetheless, the effectiveness of AADs may be incomplete and/or adverse effects may limit their use despite efficacy. Ideally, to maximize the clinical profile of AADs, clinicians should be aware of the full array of their complexities and selection options. However, commonly, some of them seem to be frequently underappreciated. Among others, these include dosing intricacies, targeting therapy to the arrhythmia trigger, avoiding habitual choices of AAD selection, consideration of AAD combinations, and more. These factors are discussed in this paper as a means of improving AAD use by clinicians and tolerance by patients. Notably, it is only these issues that are the focus of this paper, which is not meant as a review of the pharmacologic profile of each of our AADs.

## Introduction

Anti-arrhythmic drugs (AADs) have been a mainstay of dysrhythmia control for over a century. Even in the current era of increasing ablation procedures, AADs remain an important therapy. AADs may be first-line therapy, second-line therapy after failure of a prior drug(s), or added post-ablation. Nonetheless, while AADs can be effective, they are far from perfect. Effectiveness may be incomplete or absent and/or adverse effects may limit their use despite efficacy.

**Figure 1:**
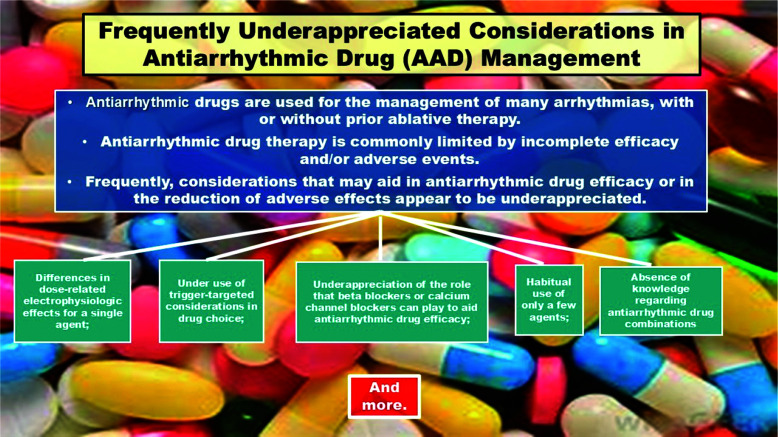
Frequently underappreciated considerations in anti-arrhythmic drug management. It presents the concerns as well as the means by which they may be addressed by clinicians who use AADs. Each of these is discussed in detail in the manuscript.

In general, the adverse effects of AAD tend to be dose-related. Thus, in many circumstances, the ability to enhance AAD effectiveness without increasing the dose would seem desirable. Notably, this may be possible more frequently than most clinicians appreciate. Electrophysiologists should know this; many cardiologists likely do too; but primary care physicians may not. Accordingly, for those who do not, one purpose of this paper is to describe several measures by which this may be accomplished. Similarly, increasing efficacy and/or decreasing adverse effects may be achieved by (1) appreciating that some AADs have different electrophysiological profiles at different doses, (2) acknowledging that patient- and arrhythmia-specific novel dosing regimens may be helpful, and (3) considering the full array of agents and formulations available rather than always turning to only a select few (such as amiodarone) **(Figure 1)**.

## Anti-arrhythmic drug–enhancing effects of β-blockers and calcium channel blockers

It has long been recognized that electrophysiological/anti-arrhythmic actions of AADs are reversible by catecholamines/sympathetic nervous system stimulation and that β-receptor blockade can counteract this effect.^1–4^ For example, in 1996, Sager and Behoodikah^1^ demonstrated that quinidine (a class I AAD) increased human ventricular refractory periods at a variety of paced cycle lengths but also that this effect could be entirely reversed by the addition of isoproterenol. However, when the experiment was repeated with *d,l*-sotalol (a class III AAD with β-blocker actions as well), the very similar prolongation of refractory periods produced with sotalol was not reversed with the subsequent addition of isoproterenol. Similarly, in a separate study, amiodarone was less affected than the investigational agent sematilide, which has no β-blocking properties.^4^ Moreover, in 2022, Wang et al.^5^ demonstrated that dronedarone’s efficacy with respect to reducing atrial fibrillation (AF) recurrence post-ablation was greater when given with metoprolol than without it. Accordingly, when an AAD is administered to patients without a contraindication to β-blockers or with a concomitant condition that might include β-blockers amongst its therapeutic options, the AAD may be more effective (and perhaps require a lower dose) if it is combined with a β-blocker.

Similarly, non-dihydropyridine calcium channel blockers may enhance AAD efficacy, particularly verapamil, which, unlike diltiazem, has some inhibitory effects on sympathetic nerve traffic and at the level of cardiac sympathetic ganglia.^6–8^ This AAD-enhancing effect has been most apparent when assessing early recurrences of AF following cardioversion. Both Tielman et al. in 1997^9^ and Koldenhof et al. in 2022^10^ demonstrated greater maintenance of sinus rhythm following cardioversion of AF when verapamil was given than in its absence, with Koldenhof et al. also showing better maintenance of sinus rhythm with verapamil than with a β-blocker when such agents were used for rate control in post-cardioversion patients. Accordingly, if desired efficacy is not achieved with an AAD, rather than increasing its dose or changing the AAD, one might consider combining it with an agent that inhibits catecholamine reversal. Clues to this consideration might include arrhythmia recurrence during exercise, stress, or stimulant use.

Finally, of relevance to β-blockers is the concomitant presence of sinus node dysfunction and AF, commonly referred to as the brady–tachy syndrome (BTS) or tachy–brady syndrome (TBS).^11–13^ In TBS, marked sinus bradycardia or sinus pauses raise concern about the ability to use a β-blocker, a calcium channel blocker, or digitalis if needed for ventricular rate control during AF in the absence of an implanted pacemaker. A related issue is the problem faced by patients whose AF has periods of both rapid and slow ventricular responses. Slowing the rapid rates without producing marked bradycardia at other times is the clinical enigma. Herein, pindolol (as an alternative to implanting a pacemaker) can be of specific value. Pindolol is a non-selective β-blocker that also possesses intrinsic sympathetic activity (ISA),^14^ more so than the alternative ISA-possessing β-blocker acebutolol. ISA provides a degree of agonistic effect at the β-receptor at rest, while the drug’s β-blocker actions exert typical β-blocker inhibitory effects during catecholamine stimulation. Accordingly, pindolol is much less likely to cause further sinus node slowing during sinus brady-rhythms,^15,16^ although it can provide ventricular rate control without further adverse slowing during AF. In 1992, Reiffel^17^ reported on 12 patients with BTS, all of whom failed to achieve ventricular rate control during AF while on digoxin, with seven having previously been shown to develop marked bradycardia (rates <40 bpm) on a standard β-blocker. In all 12 patients, the addition of pindolol led to a reduction in the mean ventricular rate to <100 bpm and the peak ventricular rate to <140 bpm without further reduction in the minimal ventricular rate observed on digoxin alone. In 11 of the 12 patients, the lowest ventricular rate on digoxin alone (<40 bpm) increased to at least 45 bpm and averaged 13 bpm higher despite continuing digoxin. These data are in keeping with those reported by James et al.^18^ in a population of similar size and demographics. Notably, data from larger AF studies are not available to support these observations. For example, in the Atrial Fibrillation Follow-up Investigation of Rhythm Management (AFFIRM) trial,^19^ one of the largest studies to date, β-blockers were used, but the specific agents were not listed. Moreover, only 5.1% of patients had drug discontinuation for bradycardia, and, while pacemakers were ultimately used in about 5% of patients, whether some patients might have avoided pacemaker implantation if pindolol had been used is entirely unknown. Nonetheless, this information regarding pindolol seems worth adding to clinicians’ knowledge base.

## Important dosing considerations

AADs are used by all physicians who treat arrhythmias. However, it is probable that not all appreciate some of the intricacies that can maximize efficacy or reduce intolerance. This paper describes three representative situations to illustrate the importance of this issue—including the use of disopyramide, the use of sotalol, and formulation selection.

### Disopyramide for nocturnal atrial fibrillation

AF episodes can be triggered by the autonomic nervous system.^20^ Such induction may occur via either sympathetic or parasympathetic actions, both of which shorten atrial refractory periods. Vagal-triggered AF may manifest as postprandial AF or nocturnal AF, whereas sympathetic-triggered AF may result from periods of heightened sympathetic tone or transmitters, such as with exercise, excitement, or mimetics, including caffeine.^21^ Autonomic-mediated AF is more likely in non-elderly patients, particularly those with paroxysmal AF,^22^ and usually has no clinically recognized trigger aside from the autonomic alteration. Therapeutically, vagal-induced AF may be particularly responsive to disopyramide.^20^ Disopyramide, a class IA AAD, has significant vagolytic actions in addition to its sodium channel–blocking effects. Quinidine does as well, but to a much lesser extent. Commonly, the vagolytic side effects of disopyramide, such as constipation, dry eye, and dry mouth, have been limiting factors in its use. However, as noted elsewhere,^23^ in patients whose AF consistently begins during sleep, disopyramide may need to be administered only prior to bedtime without another dose in the morning or during the day. This regimen can be quite effective and much better tolerated than its typical twice-a-day (bid) or four-times-a-day (qid) (formulation-specific) dosing. Vagolytic side effects are minimal with once-a-day dosing and rarely a complaint during sleep. Of note, disopyramide for vagal AF is not discussed in either the 2024 European Society of Cardiology AF guidelines^24^ or the 2023 American guidelines^25^ despite its presence in the 2001 guidelines.^9,20^ Relatedly, for adrenergic-mediated AF, β-blockers may be effective for rhythm control in addition to rate control. In contrast, β-blockers may exacerbate vagal-mediated AF, as may digitalis.

### Sotalol for atrial fibrillation

Commercially available sotalol is a mixed racemate of its *d*-isomer and *l*-isomer.^26^ Elimination is almost completely via renal excretion such that it has no active metabolites and no non-renal pharmacokinetic drug interactions. It does, however, have several recognized pharmacodynamic drug–drug interactions related to its electrophysiological properties. The mixed racemate, *d,l*-sotalol, exhibits class III AAD actions via its potassium channel-blocking properties (possessed by both isomers) as well as class II AAD actions (β-blocker), a property of only the *l*-isomer. Notably, and often underappreciated, is that sotalol’s class II and class III actions have different dose-ranging activities.^27–29^ When renal function is normal, the β-blocker effects begin with doses as low as 25 mg/day such that they are present at the 80-mg bid dose, which is the lowest dose used clinically. The β-blocking effects then increase with increasing doses but tend to plateau by 240–320 mg/day. In contrast, the class III effects only begin at 80 mg bid (where they are minimal) and then increase linearly as the total daily dose increases. Unlike the class II effects, they do not plateau within the typical dosing range. Increasing class III effects can enhance anti-arrhythmic efficacy as well as increase the proarrhythmic risk and therefore have to be considered carefully. The latter can be minimized by avoiding the use of sotalol (or other potassium channel-blocking drugs) in the presence of torsades de pointes–favoring risk factors. In the presence of renal dysfunction, sotalol clearance is reduced, and these dose-related comments should be adjusted accordingly.

Clinicians appreciating these will understand that the failure to achieve anti-arrhythmic efficacy with 80 mg of sotalol bid does not represent inefficacy; rather, it only demonstrates the failure of a moderate dose of a β-blocker, as this dose has almost no class III effects (in the absence of renal dysfunction). Moreover, if a dose of 120 mg bid does not produce untoward β-blocker side effects, it is highly unlikely that 160 mg bid would either.

Lastly, with respect to sotalol, practitioners should be aware of the differences in dose recommendations and safety parameters as per the US Food and Drug Administration (FDA) when sotalol is used for AF versus ventricular tachycardia (VT). These can be gleaned from the package inserts for VTs versus AF upon sotalol’s separate approval for these arrhythmic types (as Betapace in 1992 and as Betapace AF in 2000, respectively). The differences reflect balancing the relative risks consequent to proarrhythmic torsades de pointes versus the risks associated with AF in contrast to VTs. For VT, the typical upper daily dose is 320 mg, but the drug is approved for doses up to 640 mg/day. Additionally, its use should be avoided if hypokalemia or hypomagnesemia is present (no values specified), and the dose should be down-titrated if renal dysfunction is present to as low as a creatinine clearance of 10 mL/min as per a table in the package insert. In contrast, when approved for AF, the AF-specific package insert^30^ notes that the upper dose is 320 mg/day and states that sotalol is contraindicated if creatinine clearance is <40 mL/min or serum potassium is <4 mEq/L.

### Formulation considerations

For reasons of patient convenience/compliance, many drugs with relatively short half-lives resulting in multiple-times-a-day dosing are ultimately reformulated in a “sustained release” (SR) or “controlled release” (CR) or similarly-referred-to variations (in contrast to an “immediate release” [IR] formulation). SR formulations allow dosing to be less frequent (such as once or at most twice per day). Importantly, because SR/CR preparations release the drug for absorption more slowly than IR preparations, peak serum levels of the absorbed SR/CR versions of the drug are lower than those achieved with IR formulation. For drugs that are both metabolized by the liver and whose hepatic enzymatic process can be saturated, the slower drug release with lower peak serum levels results in a greater percentage of the drug being metabolized. Consequently, a greater total daily dose of the SR/CR preparation is required to achieve a similar amount of drug reaching the target sites in the body. Commonly, the total daily dose of an SR/CR preparation is as high as up to 50% greater than the total daily dose used for the IR preparation. For example, a 25-mg bid dose of IR carvedilol approximates the CR dose of 80 mg/day (per the package insert).^31^ In the realm of currently marketed AADs, propafenone falls into the category of having IR and SR preparations. However, the dosing interchange with the propafenone preparations varies from this general rule. This relates to the doses studied during its clinical development program rather than being based on pharmacokinetic equivalency.

In the United States, propafenone is available as an IR preparation, where dosing is suggested to begin at 150 mg three times a day (tid) and increased thereafter as needed to 225 mg tid or 300 mg tid. In the United States, the SR preparation is available as 225-, 325-, and 425-mg capsules for use bid. Thus, the peak IR dose of 300 mg tid (900 mg/day) does not approximate (is greater than) the serum levels expected with the peak dose of the SR preparation of 425 mg bid (850 mg/day). This results in two points of note for prescribing clinicians. The first is that, if the peak dose of the SR preparation is tolerated but incompletely effective, a switch to the IR preparation of 300 mg tid would not be unreasonable, as the achieved serum levels would be expectedly higher. The second is that, when propafenone is used as a pill-in-the-pocket regimen^32^ for pharmacologic cardioversion of recent-onset AF, the IR rather than the SR formulation should be used because the IR form is more rapidly absorbed and achieves greater levels.

## Additional considerations

Beyond these considerations, but as suggested in the opening comments and consistent with the theme of this paper, there are yet other underappreciated considerations that can be of importance in maximizing AAD benefits and/or minimizing their adverse side effects.

One relates to the differences between intravenous (IV) and oral versions of some of our AADs. For example, while parenteral procainamide and oral procainamide are extremely similar in their electrophysiological actions, corresponding electrocardiographic and anti-arrhythmic effects, and proarrhythmic potential, the IV formulation can be limited due to hypotension, whereas this is not an effect of the oral form. In contrast, IV amiodarone has notable differences from its oral congener. These are detailed in their respective package inserts^33,34^ but include specific instructions regarding loading regimens and a table that details differences in electrophysiological actions on multiple cardiac regions. The latter notes that, unlike oral amiodarone, IV amiodarone does not prolong the sinus cycle length, QRS duration, corrected QT interval, or the right atrial or right ventricular effective refractory period, except at doses of >10 mg/kg. Moreover, the package insert specifically states: “These differences between oral and IV administration suggest that the initial acute effects of intravenous amiodarone may be predominately focused on the AV node, causing an intranodal conduction delay and increased nodal refractoriness due to slow channel blockade (class IV activity) and noncompetitive adrenergic antagonism (class II activity).” Thus, it should not be surprising that, if IV amiodarone is given during AF, the ventricular rate will slow (typically after the first 300–400 mg), whereas, if conversion to sinus rhythm occurs, it is usually not until 1–2 g have been administered. As with IV procainamide, IV amiodarone can be limited by hypotension. Another contributor to differences between IV and oral amiodarone is the timing of the production of its clinically active metabolite, desethylamiodarone.

A second consideration in maximizing efficacy/minimizing adverse events is to consider the full array of AADs that may be used for a given arrhythmia with a given patient profile. While the major organizational guidelines^24,25^ provide highly valuable information in this respect and should be considered as first-line advice, they are only guidelines,^35,36^ and additional material from the literature, individual physician experience, and patient characteristics should also be considered. Note here that major organizational guidelines, such as those from the United States versus Europe and others, are not 100% concordant with each other. Additionally, although choosing an AAD with regulatory approval for the arrhythmia being treated should be considered, off-label use of others may be valuable. For example, amiodarone does not have FDA approval for the treatment of AF; yet, it is the most frequently prescribed AAD for AF,^37^ where prescriptions written for AF have represented up to 70% of the AADs in some surveys. In this light, practitioners should also consider ranolazine, off-label, for the treatment of AF and VTs. Its off-label status relates primarily to financial decisions by its branded manufacturer, who did not pursue FDA approval for this indication. Ranolazine was already approved and on the market for ischemic heart disease, and, by the time adequate rhythm control reports were available, generic ranolazine was on the very near horizon. There is more than ample experience to demonstrate its value and reasonably high efficacy in these settings,^38–41^ and, importantly, it is now recognized in the 2025 expert consensus statement regarding all AADs just published in *Europace*.^41^ Not only is ranolazine often effective but it rarely, if ever, causes sinus node or atrioventricular conduction dysfunction, hypotension, or proarrhythmia. In fact, ranolazine, due at least in part to its late sodium inhibition, blocked the development of torsades de pointes produced by other drugs such as dofetilide or sotalol when it was co-administered with them in well-designed laboratory studies.^41–45^

A final consideration with respect to increasing AAD efficacy/tolerance and minimizing dose levels is the use of AAD combinations. While not discussed with any strong consideration in either of the most recent American or European AF guidelines, its role in resistant arrhythmias or patients with intolerance to standard AADs in full dose should be considered. However, I will not detail this topic here, as it is amply covered in a recent review.^46^

In closing, I hope that the presented information will aid practitioners who prescribe AADs or handle patients in whom AADs have been given to best achieve the desired therapeutic goals with the least likelihood of adverse effects and greatest patient satisfaction.
